# Rapid Open-Source SNP-Based Clustering Offers an Alternative to Core Genome MLST for Outbreak Tracing in a Hospital Setting

**DOI:** 10.3389/fmicb.2021.636608

**Published:** 2021-04-01

**Authors:** Judit Szarvas, Mette Damkjaer Bartels, Henrik Westh, Ole Lund

**Affiliations:** ^1^Research Group for Genomic Epidemiology, Division for Global Surveillance, National Food Institute, Technical University of Denmark, Lyngby, Denmark; ^2^MRSA Knowledge Center, Copenhagen University Hospital Hvidovre, Hvidovre, Denmark; ^3^Department of Clinical Microbiology, Copenhagen University Hospital Hvidovre, Hvidovre, Denmark; ^4^Department of Clinical Medicine, Faculty of Health and Medical Sciences, University of Copenhagen, Copenhagen, Denmark

**Keywords:** whole-genome sequencing, surveillance, cgMLST, pathogens, nosocomial infections, MRSA

## Abstract

Traditional genotyping methods for infection control of antimicrobial-resistant bacteria in healthcare settings have been supplemented by whole-genome sequencing (WGS), often relying on a gene-based approach, e.g., core genome multilocus sequence typing (cgMLST), to cluster-related samples. In this study, we compared clusters of methicillin-resistant *Staphylococcus aureus* (MRSA) and *Enterococcus faecium* analyzed with the commercial cgMLST software Ridom SeqSphere+ and with an open-source single-nucleotide polymorphism (SNP)-based phylogenetic analysis pipeline (PAPABAC). A total of 5,655 MRSA and 2,572 *E. faecium* patient isolates, collected between 2013 and 2018, were processed. Clusters of 1,844 MRSA and 1,355 *E. faecium* isolates were compared to cgMLST results, and epidemiological data were included when available. The phylogenies inferred by the two different technologies were highly concordant, and the MRSA SNP tree re-captured known hospital-related outbreaks and epidemiologically linked samples. PAPABAC has the advantage over Ridom SeqSphere+ to generate stable, referable clusters without the need for sequence assembly, and it is a free-of-charge, open-source alternative to the commercial software.

## Introduction

The spread of antimicrobial-resistant bacteria such as methicillin-resistant *Staphylococcus aureus* (MRSA), vancomycin-resistant *Enterococcus faecium* (VRE), and carbapenemase-producing organisms (CPO) in hospitals and healthcare facilities is a growing problem worldwide. Resistant bacteria are estimated to cause the death of 33,000 people in Europe (Cassini et al., 2019) and 35,000 people in the United States (US CDC, 2019) each year. In the recent years, whole-genome sequencing (WGS) has become increasingly prevalent in routine clinical microbiology and infection control and has proven to be an excellent tool for outbreak investigations both in healthcare settings (Humphreys and Coleman, 2019; Correa-Martinez et al., 2020) and in the foodborne transmission of pathogens (Allard et al., 2018). The combination of epidemiological data and WGS makes it possible to track the transmission between patients in order to reveal an outbreak and avoid further transmission. However, genomic surveillance for infection control generates a large amount of WGS data over time, which calls for a fast and reliable tool for analysis and comparison of the genome data.

Typically, gene-based approaches, such as core genome multilocus sequence typing (cgMLST) (De Been et al., 2015; Cunningham et al., 2017; Coll et al., 2020; Gona et al., 2020; Slott Jensen et al., 2020) or whole-genome multilocus sequence typing (wgMLST) (Gateau et al., 2019), are used for subtyping isolates to detect genetic linkage of isolates. In addition, reference-based single-nucleotide polymorphism (SNP) calling (Davis et al., 2015; Katz et al., 2017; Besser et al., 2018) could be performed, which allows inferring phylogenetic relatedness and transmission routes. Although large-scale SNP analysis could be used for surveillance (Dallman et al., 2018; Szarvas et al., 2020), its performance in a hospital setting is yet to be assessed.

The purpose of this study was to compare the SNP-based open-source PAPABAC (pipeline for automated phylogenomic analysis of bacterial isolates; Szarvas et al., 2020) with cgMLST based on the commercial software Ridom SeqSphere+. The study included 5,655 MRSA patient isolates and 2,572 *E. faecium* patient isolates from Denmark, from 2013 to 2018, sequenced on Illumina MiSeq. The clustering of isolates by the two methods was compared to evaluate the performance of the SNP-based analysis. Available epidemiological data were included for the MRSA isolates to evaluate the clustering.

## Materials and Methods

### Whole-Genome Sequences

A total of 5,655 MRSA and 2,572 *E. faecium* patient isolates that were whole-genome sequenced at the Department of Clinical Microbiology at Hvidovre Hospital between January 2013 and January 2018 were included for further analysis. Sequencing runs with unknown multilocus sequence types (STs) or with differing STs in Ridom SeqSphere+ (Ridom GmbH, Germany) and in PAPABAC were excluded from the analysis.

As described before (Bartels et al., 2015; Pinholt et al., 2015), sequencing libraries were made with Nextera XT DNA sample preparation kit (Illumina, United States) after DNA concentrations were normalized with Qubit (Invitrogen, United Kingdom). Paired-end sequencing was done on MiSeq (Illumina, United States), yielding 150 base pair (bp) reads. For isolates presented in the figures, the raw sequencing data are available in BioProjects PRJEB14625, PRJEB28731, PRJEB8719, PRJNA573568, and PRJNA691722.

### Core Genome MLST and Phylogenetic Analysis

Raw reads were assembled using SPAdes (Bankevich et al., 2012). Multilocus sequence typing (MLST) and cgMLST were done with SeqSphere+ v2.3-6.0 (Ridom GmbH, Germany). Samples with low-quality assemblies were not uploaded to SeqSphere+, and in case of re-sequencing, the best-quality assembly was added to SeqSphere+. The assembly quality was evaluated by the total draft genome size, the number of contigs, and the depth of re-mapped reads. For MRSA, these are the following: the genome size is 2.6–3.1 Mbp, and the maximum number of contigs is 100. For *E. faecium*, the acceptable genome size is 2.7–3.2 Mbp, and the maximum number of contigs is 210. The minimum mean depth is 50 in all cases.

The cgMLST schemes for *S. aureus* and *E. faecium* include 1,861 and 1,423 alleles, respectively. SeqSphere+ creates clusters for *S. aureus* with a threshold of 24 or fewer allelic differences and for *E. faecium* with a threshold of 20 or fewer allelic differences.

Phylogenetic analysis was performed with PAPABAC v2.0 (Szarvas et al., 2020). In short, raw reads were matched and aligned to reference sequences of chromosomal genomes already in the PAPABAC database with k-mer identities greater than 85.15%, creating equal-length consensus sequences for each sample. These were gathered for each genome, and SNP-based distances were calculated between them. Sequencing runs that had more than 12% of unknown bases in their consensus sequences were excluded from the analysis. The threshold of 12% was based on empirical data analysis over the whole dataset for this study and isolates available in Evergreen Online. To filter low-quality regions from the alignment, all columns were removed from the sequence alignment that had one or more unknown base up until 100 non-redundant runs in the set or until the number of columns that needed to be removed exceeded 25% of the total length of the consensus sequence. Thereafter, all columns that had fewer than 90% known bases in them were removed. To reduce the computational time for calculating the genetic distance and inferring phylogeny on the growing dataset, sequences were de-replicated with a threshold of 10 SNPs, creating technical clusters, with the aim of reducing computational time. The distance-based clustering method was unweighted pair group method with arithmetic mean. Sequencing runs were batched according to the date of sequencing and processed chronologically. The code is available from https://bitbucket.org/genomicepidemiology/evergreen/src/COMPARE/.

Methicillin-resistant *S. aureus* samples of ST97 and single-locus variants (SLV) of ST97 were subjected to additional analysis with PAPABAC using an MRSA reference genome of ST97 from a well-described hospital outbreak in Copenhagen (Rubin et al., 2018). This genome was closed with a combination of Nanopore sequencing and Illumina MiSeq sequencing and yielded a genome of 2,781,777 bps and a plasmid of 20,403 bps. A maximum-likelihood phylogeny was also inferred with IQ-TREE v1.6.12 (Nguyen et al., 2015) with GTR + I + G nucleotide substitution model on the alignment made from the equal-length consensus sequences.

### Comparison of cgMLST and PAPABAC Results

For MRSA isolates, the clusters generated by cgMLST were compared with the clustering by PAPABAC. Some of the MRSA isolates were known to be from well-described hospital outbreaks, and for many of the other isolates, it was known whether the isolates were from persons from the same households or if they were epidemiologically connected in other ways. We did the comparison on the template set for the MRSA ST80 reference sequence *S. aureus* subsp. *aureus 11819-97* (accession: NC_017351.1) that matched the highest number of MRSA samples out of the 19 available *S. aureus* templates. Clusters of diverse traditional MLST STs were located in the dendrogram and the minimum spanning trees from SeqSphere+, and the clustering of samples were compared together with the available epidemiological information. This was done manually looking at sample identifiers from each cluster as well as singletons in the SNP tree and evaluating whether the same samples clustered together or were singletons by cgMLST. Furthermore, available epidemiological data were included if a discrepancy between the two methods was seen.

For the *E. faecium* samples, there were four templates in the database, and we used the VRE ST203 reference sequence *E. faecium Aus0085* (accession: NC_021994.1) for comparison. Epidemiological data were not included for VRE samples, but clustering was compared as described for MRSA.

Due to the assembly or consensus sequence quality requirements for inclusion, samples might not be present in both systems.

Because cgMLST creates clusters for *S. aureus* at 24 or fewer allelic differences (Coll et al., 2020) and for *E. faecium* at 20 or fewer allelic differences (Leopold et al., 2014; De Been et al., 2015), we considered sequences with the same SNP thresholds to be likely genetically related during the evaluation of the PAPABAC trees.

Clustering congruence was estimated for both organisms on the shared samples after re-clustering the distance matrices with the clustering thresholds stated above using average-linkage hierarchical clustering, calculating the adjusted Rand index (Hubert and Arabie, 1985) and the Wallace coefficient (Wallace, 1983) using the partitionComparison v0.2.5 R package. Moreover, Spearman’s rank correlation coefficient was calculated on the distance matrices containing only the non-redundant samples from PAPABAC.

## Results

### MRSA Outbreaks and Other Epidemiologically Connected Samples

Between January 2013 and January 2018, 5,655 sequencing runs were generated from DNA extracted from MRSA isolates. Out of these, 1,930 genomes were matched to the reference sequence *S. aureus* subsp. *aureus 11819-97*, and after discarding low-coverage sequences, 1,848 genomes were included in the last phylogenetic tree (Supplementary Data 1). A total of 1,804 genomes were *in silico* predicted to contain the *mec*A gene. The genomes in the phylogenetic tree belonged to 39 traditional MLST STs. The most abundant ST was ST6, followed by ST1 and ST8.

There were 630 genomes that did not cluster to another at the 10 SNPs technical threshold, while 1,218 genomes clustered in 352 technical clusters. Technical clusters have a median size of two and a mean size of 3.46 genomes. Looking at the distribution of STs in these 352 clusters, there were seven (2.0%) that also contained at least one genome that was an SLV of the ST of the cluster.

On systematic comparison between clustered samples with 24 allelic or SNP difference threshold, the adjusted Rand index was 0.60, and the Wallace coefficients were *W*_PAPABAC⟶SeqSphere+_ = 0.996 and *W*_SeqSphere+⟶PAPABAC_ = 0.434. Spearman’s rank correlation coefficient for the distance matrices was found to be 0.83.

**FIGURE 1:**
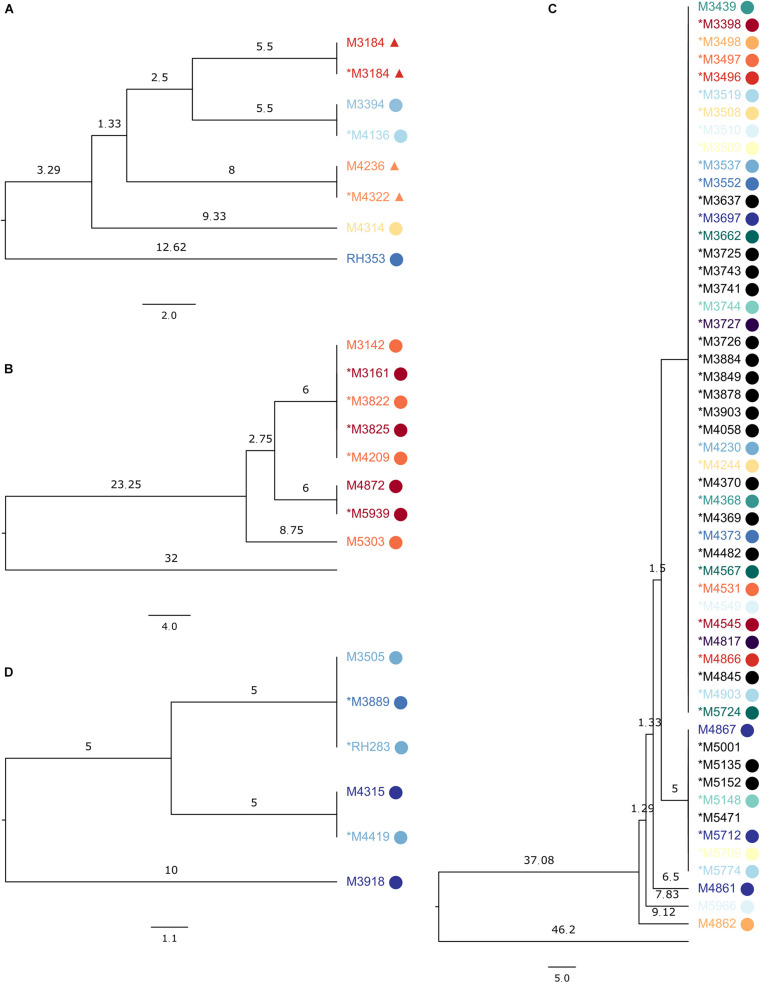
Subsections of the UPGMA tree made on the reference sequence *Staphylococcus aureus* subsp. *aureus* 11819-97, showing SeqSphere+ clusters, with samples from the same patient colored the same and epidemiologically related samples displaying the same symbol. The asterisk denotes samples in the technical clusters. Branch lengths are single nucleotide differences. **(A)** ST1 clusters with seven samples; the sequences clustered were from the same biological sample or from patients sharing a household (labels marked with circle or triangle) or from the same patient. **(B)** ST1 cluster with eight sequences from the same household. **(C)** ST1 cluster with 51 sequences from members of connected families and unrelated individuals. **(D)** ST852 cluster with six sequences from the same household.

Some examples of MRSA SNP clusters are shown in Figure 1. One ST1 cluster (Figure 1A) contained seven genomes in the SNP-based trees, with a maximum SNP distance of 18, while the maximum allelic distance was 13 in SeqSphere+ (Supplementary Figure 1A). The technical clustering connected sequencing runs of the same sample, temporally displaced samples from the same patient, and samples from epidemiologically related patients. Another cluster with eight samples of a maximum allelic difference of seven in SeqSphere+ (Supplementary Figure 1B) presented in our tree with a maximum distance of 17 SNPs (Figure 1B), encompassing one technical cluster of five isolates from two patients from the same household, another one with two isolates from the same patient from this household, and a singleton sample from the other patient. The last cluster of ST1 that we inspected had 51 samples in the SNP tree, divided over two clades and three additional singletons, with a maximum distance of 18 SNPs. In SeqSphere+, the maximum allelic difference was 15. The samples were from members from four connected families and two unrelated individuals (Figure 1C).

Eleven ST1835 samples formed a cluster of a previously described outbreak (Ramsing et al., 2013) with both clustering technologies (Supplementary Figure 2). For ST852, six samples from a household clustered together in SeqSphere+ with a maximum allelic difference of 11, while our tree placed them within 20 SNPs but into two clades and one sample as a singleton (Figure 1D).

**FIGURE 2:**
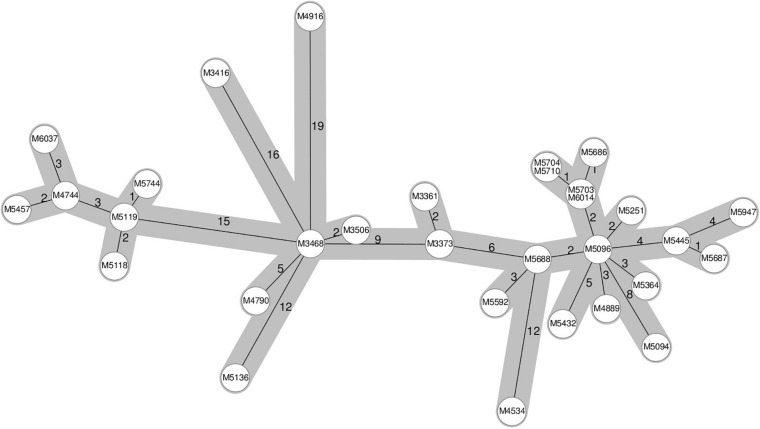
Ridom SeqSphere+ cgMLST minimum spanning tree of a 5-year-long hospital-related outbreak. The numbers on the edges between the nodes denote the allelic distances between samples.

**FIGURE 3:**
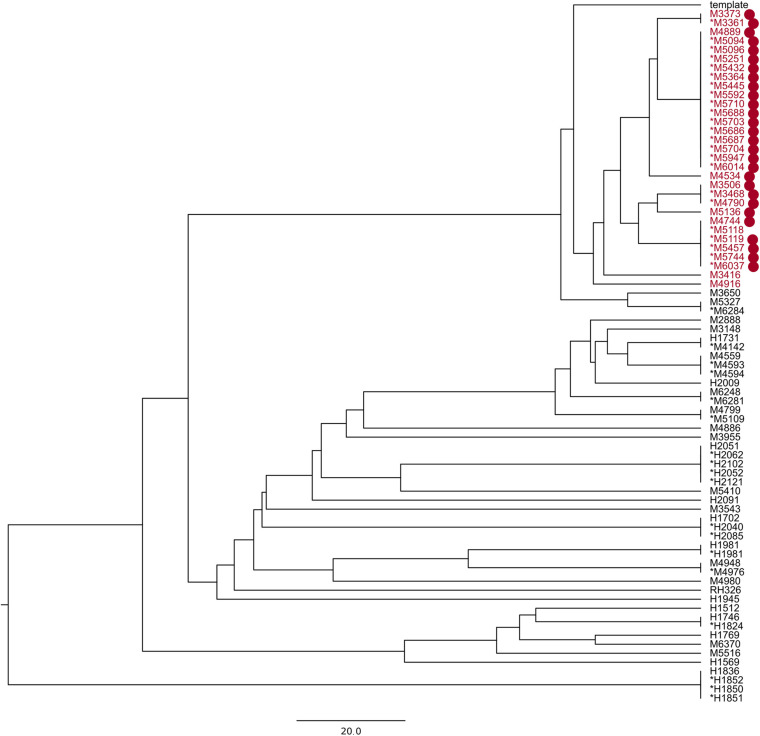
UPGMA phylogenetic tree made with reference *Staphylococcus aureus* M3261; the samples in red are part of a known hospital outbreak, with supporting epidemiological data denoted with red dots. The asterisk denotes samples in the technical clusters. The scale bar is the equivalent of 20 SNPs.

A known hospital outbreak of ST97 MRSA (Rubin et al., 2018) spanning 5 years had only 22 isolates in the SNP tree as the remaining nine isolates related to the outbreak did not have the required identity to the reference sequence *S. aureus* subsp. *aureus 11819-97*, and they were assigned to a different reference, thereby splitting the outbreak isolates. The 22 outbreak isolates in the SNP tree were situated across four technical clusters with a distance of 42 SNPs. We repeated the analysis of all ST97 samples from the database (*N* = 79) with a closed reference genome from this particular outbreak (Supplementary Data 2). All 31 outbreak samples (Figure 2) were now assigned to the outbreak reference genome but were divided into four technical clusters and four singletons within 40 SNPs (Figure 3).

### *E. faecium* Clusters

A total of 2,572 sequencing runs were produced between March 2013 and January 2018 that were classified to be *E. faecium*. The reference sequence *E. faecium Aus0085* collected 1,755 samples, of which 1,355 genomes had the necessary coverage to be included in the phylogenetic tree (Supplementary Data 3). These genomes were distributed over 17 STs, with ST203, ST117, and ST80 being the most abundant STs. Then, 1,247 genomes were clustered into 89 technical clusters with the 10 SNPs distance threshold. The median size of these was three genomes, and the mean size was 13.27 genomes. Eighty-three clusters contained just one ST, and six clusters (6.7%) contained one or more genomes being SLVs of the dominant ST of the cluster.

The adjusted Rand index of the clusters with the threshold of 20 allelic or SNP differences was calculated to be 0.80. Meanwhile, the Wallace coefficients were *W*_PAPABAC⟶SeqSphere+_ = 0.995 and *W*_SeqSphere+⟶PAPABAC_ = 0.721. Spearman’s rank correlation between the distance matrices was 0.78.

We compared SNP-based clusters that were larger than two sequences to SeqSphere + cgMLST clusters. All samples that were clustered with PAPABAC also clustered together *via* cgMLST (Supplementary Figure 3).

## Discussion

Surveillance and infection control in hospitals that embrace WGS require a system for processing and comparing thousands of sequences. Gene-based solutions have become popular as these allow for all-against-all comparisons with a common nomenclature. They achieve this by reducing the number of features in the comparison to only a few thousand gene alleles instead of millions of bps per samples. However, these methods require an available scheme for the species in question, with a constantly updated database of alleles for each locus and correct *de novo* assembly of the WGS data (Leopold et al., 2014). *De novo* assembly is a computationally complex problem that requires a long run time, measured in hours on a single thread (Bankevich et al., 2012). Moreover, for high-quality assemblies that could be used in gene-based subtyping, error correction of the draft assembly by mapping the reads to the assembly is also necessary if the *de novo* assembler does not perform it by default (Walker et al., 2014). Conversely, reference-assisted assembly of WGS reads could be done in minutes (Li, 2013, 2018), and the high-quality SNPs or consensus sequences thereafter could be used in an SNP-based analysis. We previously demonstrated a method for large-scale SNP-based phylogenetic analysis to be accurate from a few dozens to several hundred samples of food-borne Gram-negative bacteria. The stand-alone pipeline, PAPABAC, is available as open-source software. It can run automatically on new-input raw sequencing data, quickly generating stable clusters of closely related isolates, making it ideal for use in continuous genomic surveillance. The clusters can be referred to by the sample identifier of the cluster representative, i.e., the first isolate in the cluster, and the template name. Moreover, it can accommodate the growing number of samples without a significant increase in CPU time (Szarvas et al., 2020).

Incorporating enhanced filtering for uncertain bases, we applied PAPABAC to Gram-positive bacteria that are relevant in nosocomial infections, namely, *S. aureus* and *E. faecium*. After the last batch had been included for analysis in this study, the resulting clusters produced by the 10 SNPs technical threshold encompassed sequences with the same STs or, in seven cases for MRSA and six cases for *E. faecium*, only differed in one SNP in one locus, indicating a good specificity for related sequences.

For a systematic comparison of the two different methods, the distance matrices were re-clustered at the appropriate 24 or 20 allelic or SNP differences, and the congruence between them was assessed with adjusted Rand and Wallace clustering coefficients. Adjusted Rand indices of 0.60 and 0.80, for MRSA and *E. faecium*, respectively, indicate correlation between the clustering that is not due to random chance, with the agreement between clusters for *E. faecium* being higher than for MRSA. The Wallace clustering coefficients for the PAPABAC clusters were larger for both organisms, indicating that the PAPABAC clustered samples also clustered together in cgMLST, but the samples clustered with the same threshold in cgMLST do not necessarily cluster with PAPABAC. This is the consequence of the SNP-based method having a higher discriminatory power than the gene-based cgMLST. Recently, genetic relatedness cutoff values for excluding short-term transmission have been published for cgMLST, wgMLST, and core SNP methods for MRSA (Coll et al., 2020). Similarly, cutoff values for SNP-based methods should be estimated for *E. faecium*.

Spearman’s rank correlation coefficients were computed to compare the distance matrices themselves, and we obtained coefficients of 0.83 and 0.78 between the two distance methods, the imperfect correlation explained by the different resolutions provided by the two methods.

Sub-trees of the phylogenetic trees produced by PAPABAC were also compared to the cgMLST minimum spanning trees generated with SeqSphere+, and for the MRSA samples, the available epidemiological data were also used to evaluate the clustering. Although we could not compare all clusters in this manner due to their numbers, we expected a similar level of concordance in the rest of the clusters. We found that there was a general agreement between the two methods as to what sequences could have common origins or be part of an outbreak if we designate 24 allelic or SNP differences or fewer for MRSA and 20 allelic or SNP differences or fewer for *E. faecium* to indicate this. We identified known MRSA outbreaks (Figures 1C, 3 and Supplementary Figure 2) and patients who were close contacts (Figures 1A,B,D). However, epidemiological data could not support the links between all samples in some clusters, like in Figure 1A, yet it remained true that epidemiologically connected samples were closely related genetically.

Choosing the correct reference genome is important when using an SNP-based analysis (Besser et al., 2019; Uelze et al., 2020). This is highlighted by the MRSA ST97 outbreak presented here, where some sequences were more similar to a different reference sequence in the default database, splitting the outbreak between two phylogenetic trees. One solution to avoid this is to decrease the identity threshold for matching sequencing runs to reference sequences. However, low identity sequences would decrease the alignment length, leading to apparently higher similarities between sequences. A better solution is to use in-house reference genomes that match closely with the pathogens present in a given population and location. We found that, aligning to the in-house ST97 reference genome, all of our outbreak-related samples could be placed onto the same phylogenetic tree, with 40 SNPs between them at most. This is larger than what is usually considered to be closely related (De Been et al., 2015) but can be explained by the fact that the outbreak isolates were spanning over a 5-year period. Using one of the outbreak isolates as reference genome for the SNP-based method gave a marginally higher resolution between the genomes in the outbreak than using a more distant reference genome. CgMLST schemes are only available for a number of bacteria, and profiles available in SeqSphere+ are maintained and updated through the SeqSphere+ software. New alleles in the query sequence(s) are not included in the distance calculation unless submitted to cgMLST.org. Local cgMLST schemes could be defined; however, it is a laborious task compared to selecting a suitable reference genome.

In short, the two methods yield concordant phylogenies, and PAPABAC has the advantage over Ridom SeqSphere+ to work with very large datasets and generate fast, stable, and referable clusters without the need for sequence assembly, and it is a free-of-charge open-source alternative to the commercial software.

## Data Availability Statement

The data presented in the study are deposited in the NCBI SRA repository, accession numbers PRJEB14625, PRJEB28731, PRJEB8719, PRJNA573568, and PRJNA691722.

## Author Contributions

OL and HW conceived the study. JS, MB, OL, and HW designed the study. JS developed the code and analyzed the data. JS and MB evaluated the results and wrote the manuscript with input from all the authors.

## Conflict of Interest

The authors declare that the research was conducted in the absence of any commercial or financial relationships that could be construed as a potential conflict of interest.
